# Pediatric otosclerosis: Case report and literature review

**DOI:** 10.1016/S1808-8694(15)31105-8

**Published:** 2015-10-19

**Authors:** Raquel Salomone, Paulo Emmanuel Riskalla, Andy de Oliveira Vicente, Maria Carmela Cundari Boccalini, Adriana Gonzaga Chaves, Renata Lopes, Gilberto Bolivar Felin Filho

**Affiliations:** 1 Medical resident in otorhinolaryngology, CEMA Hospital; 2 Master's degree, adjunct professor in Otorhinolaryngology, UNIFESP/EPM. Coordinator and tutor of the medical residency program in otorhinolaryngology, CEMA Hospital; 3 Master's degree, doctoral student in otorhinolaryngology, UNIFESP/EPM. Tutor in the medical residency program in otorhinolaryngology, CEMA Hospital; 4 Otorhinolaryngologist, tutor in the medical residency program in otorhinolaryngology, CEMA Hospital and the City Public Servant Hospital (Hospital do Servidor Publico Municipal); 5 Graduate student; 6 Otorhinolaryngologist; 7 Medical resident in otorhinolaryngology

**Keywords:** childhood, otospongiosis, hearing loss

## Abstract

Otospongiosis is an osteodystrophy of the temporal bone, characterized by disordered neoformation and deposition of bone, characterized by the presence of a progressive conductive, sensorineural or mixed hearing loss and tinnitus. Typically, otospongiosis presents as a slowly progressive conductive hearing loss in the third to fourth decade of life. Uncommonly children and adolescents may also have conductive or sensorineural hearing loss caused by otosclerosis. We describe a case of an 11-year-old patient, with progressive unilateral conductive hearing loss for 5 years. The otoscopic examination revealed a positive Schwartz's sign in the left ear. Audiometry, impedanciometry and CT scan showed characteristics that suggested otospongiosis. We reviewed clinical aspects, diagnosis and the therapeutic approach for otospongiosis in children.

## INTRODUCTION

Otospongiosis is osteodystrophy of the temporal bone, characterized by disordered bone resorption and neoformation in genetically predisposed individuals.1 All of the otic capsule may be involved, although the area close to the fissula ante fenestram (anterior to the oval window) is the most commonly affected site.^1^^,^^2^

The incidence of otospongiosis is 7 to 10% in the general population; the onset is usually around the 3rd or 4th decades of life,^3^ predominating in Caucasians and in females (2:1 proportion relative to males). The prevalence varies from 3 to 12% in its histological form (asymptomatic), and 0.1 to 1% in its clinical form (symptomatic).^1^

This disease is autossomic dominant, with a 20 to 40% penetrance.^3^, ^4^, ^5^ In 70 to 90% of cases the disease is bilateral, as mentioned with a higher prevalence in Caucasians.^1^^,^^6^ It is infrequent and difficult to diagnose in infancy.

Clinically, otospongiosis is characterized by progressive conductive and/or mixed hearing loss and by tinnitus. Sensorineural hearing loss, aural fullness and vertigo may eventually ensue. A clinical picture similar to endolymphatic hydrops may be seen in severe cases. The onset of auditory symptoms usually is between ages 20 and 30 years.

The diagnosis is made based on the clinical history, the physical examination, and tests such as pure tone audiometry, voice audiometry and immitance testing. Image tests may also provide relevant diagnostic information; computed tomography (CT) is the preferred method.

The treatment may be medical (anti-enzyme or anti-bone remodeling drugs) or surgical (stapedotomy or stapedectomy). Personal sound amplification devices (PSAD) are a further option, particularly in patients with surgical contraindications.

The aim of this study was to report a case of child otospongiosis and to review the literature on this theme.

## REVIEW OF THE LITERATURE

Although otospongiosis is considered a disease of young adults, hearing loss may begin in the first few years of life. 10 Niedermeyer et al. reported signs of this disease in children under age 6 years.^1^^,^^6^ Guild et al. found otosclerosis in 0.6% of temporal bones in children under age 5 years, and in 4% of children aged between 5 and 18 years.^4^ North-American statistics suggest that otospongiosis is the main cause of acquired hearing loss, occurring in 15 million people.^5^ Robinson reviewed 4,014 stapedotomy cases and found that hearing loss had begun before age 18 years in 15% of the sample.^10^^,^^11^

High resolution computed tomography of the temporal bone - which is the image test of choice in this condition - makes it possible to detect abnormalities in the oval window or close to it in 90% of cases with surgical proof of the disease.^6^, ^7^, ^8^ In many cases, the final diagnosis is only possible in post-mortem histological studies of the temporal bone.^1^^,^^9^

The differential diagnosis is made with otitis media with effusion, congenital fixation of the stapes, disarticulation of the middle ear bones, tympanosclerosis, osteogenesis imperfecta, Paget's disease and congenital cholesteatoma.^6^

## CASE REPORT

A white male patient, aged 11 years, presented at our unit complaining of unilateral progressive hearing loss for 5 years, and moderate, intermittent, whistle-like tinnitus; there were no vestibular symptoms. The patient reported repeated episodes of otitis media with effusion during the past 5 years that was resistant to the usual medical treatment. The patient had a family history of otospongiosis (mother and maternal grandmother).

**Figure 1:**
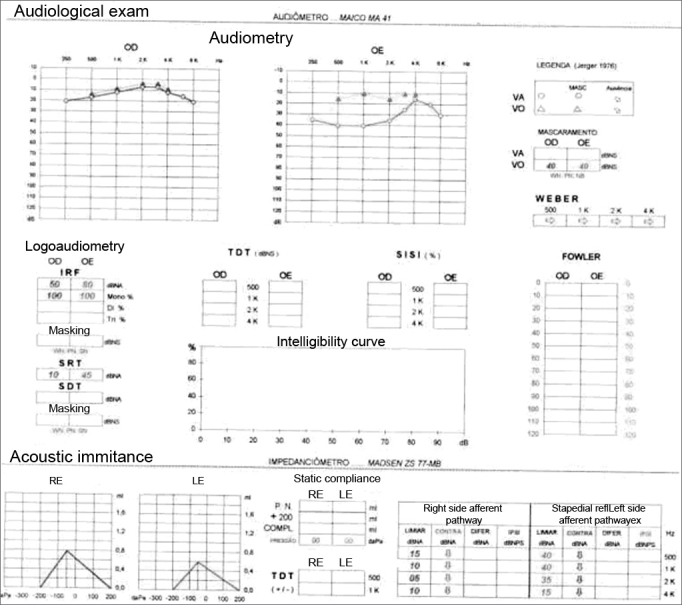
Pure tone audiometry revealing mild conductive hearing loss in the left ear. Immitance testing showing reduced left tympanic membrane complacency and bilateral absence of the stapedial reflex.

The otorhinolaryngological exam revealed a reddish hue seen by transparency through the tympanic membrane in the promontory of the left ear (Schwartze's sign) and no right ear alterations. Pure tone audiometry showed mild left ear conductive hearing loss; the right ear hearing thresholds were within normal limits. Immitance testing revealed decreased left tympanic membrane complacency and bilateral absence of the stapedian reflex (Figure 1).

**Figure 2:**
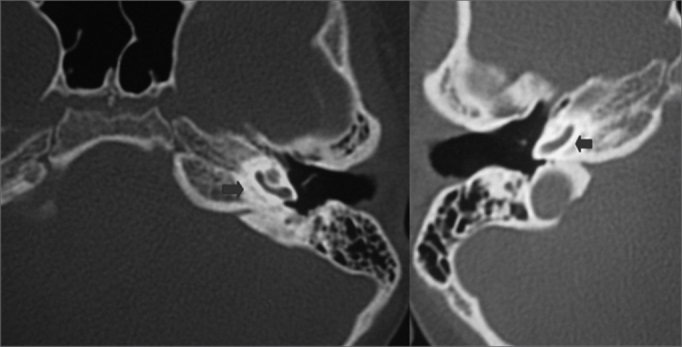
Computed tomography (CT) of the temporal bones; axial slices along the round window showing pericochlear hypodensity (otosclerosis) (blue arrows).

**Figure 3:**
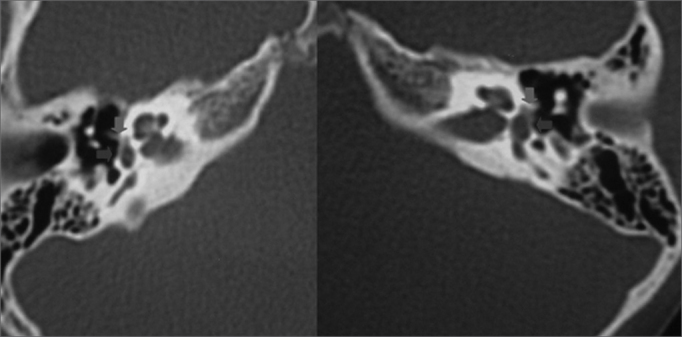
CT of the temporal bones in axial sections showing a small hypodensity in the area anterior to the oval window (green arrows) and thickening of the stapes footplate (blue arrows).

**Figure 4:**
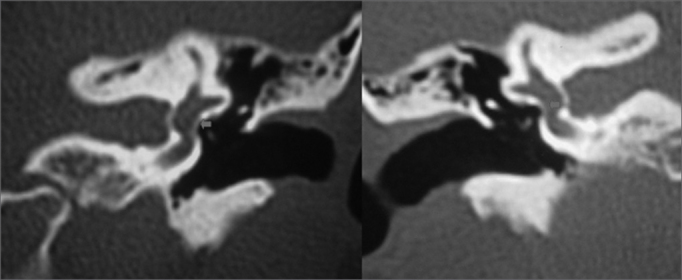
CT of the temporal bones in coronal slices along the oval window showing thickening of the stapes footplate (red arrows).

Computed tomography of the temporal bones showed anterior otosclerosis in the right and left oval windows (Figure 2, Figure 3), bilateral cochlear pericochlear foci with no endosteal involvement, and thickening of the stapes footplate to the left (Figure 4).

The clinical picture associated with Schwartze's sign and the CT findings suggest active otospongiosis.

Medical treatment with sodium fluoride (10 mg/day) was started, but the patient developed pharmacodermy, which required interrupting the use of this drug.

Surgery (stapedotomy) and a hearing aid were suggested. A choice was made for waiting and programmed outpatient monitoring (pure tone audiometry, voice audiometry and immitance testing) every six months.

## DISCUSSION

Conductive hearing loss is frequent in infancy; they are usually secondary to acute or chronic otitis media with or with no effusion.^4^^,^^10^ Other congenital or acquired causes of conductive hearing loss, such as otospongiosis and tympanosclerosis, are generally underdiagnosed or diagnosed late, which may result in delayed language development.^4^^,^^10^

A complete clinical history (including any personal or family history of hearing loss), an otorhinolaryngological examination and complementary tests are essential for the diagnosis of otospongiosis.

In our patient, we found that temporal bone CT contributed significantly to the diagnosis, given that the clinical picture and serial audiometries suggested otitis media with effusion.

Treatment approaches to otospongiosis include anti-enzyme or bone resorption moderating drugs, PSAD, and surgery. Surgery is not recommended in children below age 5 years; in such cases, PSAD are the treatment of choice.^4^ Stapedotomy is controversial after age 5 years.^10^ Some authors have argued in favor of using this procedure early, while others contend that an expectant approach should be used until adolescence.^10^^,^^12^ Cole, House and Robinson have stated that stapedotomy is a safe and effective treatment in pediatric patients.^6^^,^^11^^,^^13^^,^^14^ Various studies have shown that stapedotomy adequately treats conductive hearing loss in up to 90% of these patients.^3^^,^^10^^,^^12^^,^^15^

Our patient was treated initially with sodium fluoride (10 mg/day); the drug was stopped two days later due to a skin hypersensitivity reaction. Alendronate was not indicated due to the patient's age and the potential risk for bone development. After a careful appraisal of the case and talks with the patient's family, we decided for an expectant approach and outpatient monitoring.

## FINAL COMMENTS

A high rate of suspicion is essential for an early diagnosis of infant otosclerosis, making it possible to monitor the disease and to apply the most appropriate treatment. In this study, we found that CT of the temporal bones was extremely important in defining the diagnosis; other tests suggested persistent and/or drug-resistant otitis media with effusion.
